# GnRH—Gonadotropes Interactions Revealed by Pituitary Single-cell Transcriptomics in Zebrafish

**DOI:** 10.1210/endocr/bqae151

**Published:** 2024-11-06

**Authors:** Sakura Tanaka, Yang Yu, Berta Levavi-Sivan, Nilli Zmora, Yonathan Zohar

**Affiliations:** Institute of Marine & Environmental Technology, Department of Marine Biotechnology, University of Maryland Baltimore County, Baltimore, MD 21202, USA; Institute of Marine & Environmental Technology, Department of Marine Biotechnology, University of Maryland Baltimore County, Baltimore, MD 21202, USA; Department of Animal Sciences, The Robert H. Smith Faculty of Agriculture, Food, and Environment, The Hebrew University of Jerusalem, Rehovot 76100, Israel; Institute of Marine & Environmental Technology, Department of Marine Biotechnology, University of Maryland Baltimore County, Baltimore, MD 21202, USA; Institute of Marine & Environmental Technology, Department of Marine Biotechnology, University of Maryland Baltimore County, Baltimore, MD 21202, USA

**Keywords:** GnRH, LH, reproduction, pituitary, transcriptomics, teleost

## Abstract

GnRH governs reproduction by regulating pituitary gonadotropins. Unlike most vertebrates, *gnrh*^−/−^ zebrafish are fertile. To elucidate the role of the hypophysiotropic-Gnrh3 and other mechanisms regulating pituitary gonadotropes, we profiled the gene expression of all individual pituitary cells of wild-type and *gnrh3*^−/−^ adult female zebrafish. The single-cell RNA sequencing showed that LH and FSH gonadotropes express the 2 gonadotropin beta subunits with a ratio of 140:1 (*lhb*:*fshb*) and 4:1 (*fshb*:*lhb*), respectively. Lh gonadotropes predominantly express genes encoding receptors for GnRH (*gnrhr2*), thyroid hormone, estrogen, and steroidogenic factor 1. No GnRH receptor transcript was enriched in FSH gonadotropes. Instead, cholecystokinin receptor-b and galanin receptor-1b transcripts were enriched in these cells. The loss of the *Gnrh3* gene in *gnrh3^−/−^* zebrafish resulted in downregulation of *fshb* in LH gonadotropes and upregulation of pituitary hormones like TSH, GH, prolactin, and proopiomelanocortin-a. Likewise, targeted chemogenetic ablation of Gnrh3 neurons led to a decrease in the number of *fshb*+, *lhb* + and *fshb*+/*lhb* + cells. Our studies suggest that Gnrh3 directly acts on LH gonadotropes through Gnrhr2, but the outcome of this interaction is still unknown. Gnrh3 also regulates *fshb* expression in both gonadotropes, most likely via a non-GnRH receptor route. Altogether, while LH secretion and synthesis are likely regulated in a GnRH-independent manner, Gnrh3 seems to play a role in the cellular organization of the pituitary. Moreover, the coexpression of *lhb* and *fshb* in both gonadotropes provides a possible explanation as to why *gnrh3^−/−^* zebrafish are fertile.

The pituitary is the master endocrine regulator in vertebrates, which controls various crucial physiological processes such as growth, metabolism, stress response, osmoregulation, and reproductive functions, including ovulation. This gland is under the control of neuropeptides produced by the hypothalamus in the brain and peripheral hormones like steroids, glucocorticoid, thyroid hormone, and others.

The reproductive hypothalamus-pituitary-gonadal axis in all vertebrates is governed by the hypothalamic GnRH receptor 1 (GnRH1), which stimulates the synthesis and secretion of the 2 gonadotropins FSH and LH in and from the pituitary gonadotropes. In general, FSH induces the initial stages of folliculogenesis while pulsatile LH plays a vital role in the later phase, including final oocyte maturation and ovulation (1-3). This delicate balance of circulatory gonadotropin milieu is regulated by gonadal sex-steroid feedback to modulate the various processes of reproduction. Generally in vertebrates, loss of the hypophysiotropic GnRH leads to infertility due to reduced/insufficient gonadotropin production and secretion and the required preovulatory GnRH-LH surge in females (4-6).

Zebrafish are believed to have lost Gnrh1 during the evolution of the ostariophysi/cypriniform lineage and possess 2 isoforms: the largely species-abundant Gnrh2 and the teleost-specific Gnrh3 (7). In zebrafish, Gnrh3 neurons populate the olfactory bulbs and telencephalon as seen in all teleost species but also the hypothalamic preoptic area, which heavily projects to the proximal par distalis of the pituitary (7, 8). Hence, Gnrh3 has been suggested to fulfill the role of Gnrh1 as a master regulator for LH synthesis and secretion. However, the loss of function of *Gnrh3* in zebrafish and both Gnrh isoforms in *gnrh3*^−/−^ and *gnrh2*^−/−^; *gnrh3*^−/−^ mutant lines does not affect reproductive physiology, including oocyte maturation, ovulation, spawning, and fertility (9, 10). Furthermore, targeted chemogenetic ablation of Gnrh3 neurons in adult females results in normal reproductive fertility with no effects on plasma LH levels (11). These findings indicate that gonadotropin synthesis and secretion is regulated by a GnRH-independent manner in zebrafish.

We therefore sought to determine if the female zebrafish pituitary possesses unique cellular and/or molecular features that enable gonadotropin synthesis/release in a GnRH-independent manner and to establish a functional role for Gnrh3 at the pituitary level in this species. To achieve that, we examined gene expression profiles of individual pituitary cells of sexually mature wild-type (WT) and *gnrh3*^−/−^ female zebrafish by single-cell RNA sequencing (scRNAseq) and successfully transcriptionally characterized the various known pituitary cell types in both genotypes.

We found that *gnrhr2* is uniquely expressed in LH gonadotropes but not in FSH gonadotropes, suggesting that Gnrh3 acts on LH gonadotropes through this receptor. In addition, genes encoding estrogen receptor type 2 were enriched in LH gonadotropes. FSH gonadotropes expressed 2 neuropeptide receptors: galanin receptor 1b (*gal1rb*) and cholecystokinin receptor b (*cckbr*). The expression of most receptors and transcription factors (TFs) encoding genes in LH gonadotropes were comparable between WT and *gnrh3*^−/−^, except for *fshb* expression that was downregulated in *gnrh3*^−/−^ LH gonadotropes. Similarly, targeted ablation of Gnrh3 neurons in adult females decreased the number of *fsh^+^* cells and *fsh^+^/lhb^+^* cells. Our findings suggest that while the exact effects of Gnrh3 through Gnrhr2 in LH gonadotropes are still unknown, Gnrh3 and its neurons seem to promote *fshb* expression in LH and FSH gonadotropes through an unknown pathway, thus contributing to the cellular organization of the pituitary. These findings support the idea that Gnrh3 is functionally different from Gnrh1.

## Material and Methods

### Animal Husbandry

All zebrafish were maintained in a 28 °C recirculating aquaculture system under a 14-hour light and 10-hour dark photoperiod. *gnrh3*^−/−^ zebrafish were obtained from a previous study (10, 11). Before tissue sampling, fish were deeply anesthetized in tricaine methanesulfonate (MS-222; #E10521, Sigma-Aldrich, St. Louis, MO, USA) and rapidly decapitated. All experimental protocols were approved by the Institutional Animal Care and Use Committee at the University of Maryland School of Medicine.

### Pituitary Cell Dissociation

A week before the dissection for pituitary dissociation, 3-month-old *gnrh3*^−/−^ and their sibling *gnrh3*^+/+^ WT mature female zebrafish were crossed with WT males, and the females with 90% fertilization rate (number of fertilized eggs/number of total released eggs per spawning × 100) and ≥ 200 fertilized eggs were selected as sexually mature animals and used for further experiments. Five pituitaries were dissected from each *gnrh3*^−/−^ (body weight = 580 ± 24 mg) and WT (body weight = 547 ± 20 mg) females at 8 00 hours, which was 1 hour before lights on. The dissected pituitaries were combined by genotype into separate 1.5 mL Eppendorf tubes filled with sterile PBS and left on ice until processing. Pituitary dissociation was performed by following previously described protocols (12, 13) with some modifications. Briefly, the samples were centrifuged at 2000 × rpm for 5 minutes at 4 °C, then PBS was removed followed by a treatment of dissociation solution [0.25% Trypsin (#15090-046, Gibco, Waltham, MA, USA); 1 mM EDTA; 0.2% collagenase (#C0130, Sigma-Aldrich) in PBS; filtered with a 0.22 µm membrane filter] at 28.5 °C for 40 minutes, with gentle pipetting every 10 minutes using a wide bore tip. After incubation, the treatment was stopped by adding 200 µL 6×Stop solution (6 mM CaCl_2_; 30% fetal bovine serum in PBS) and gently mixed it by pipetting, followed by centrifuging at 2000 × rpm for 5 minutes at 4 °C. The supernatant was carefully discarded, then 1 mL Accumax working solution [30% Accumax (#07921, STEMCELL Technologies, Vancouver, Canada) in PBS] was added, and the cells were gently resuspended. After incubation at room temperature for 5 minutes, the dissociated cells were filtered through 40 µm pore cell strainer (#H13680-0040, Flowmi Cell Strainers; Bel-Art, Wayne, NJ, USA). After centrifuging at 2000 × rpm for 5 minutes at 4 °C, the supernatant was removed, and the cell pellets were gently resuspended with 50 µL 0.1% BSA in PBS and kept on ice until processing. Cell viability was assessed by mixing the previous suspension with trypan blue dye and counting the number of live and dead cells using an automated cell counter (Bio-Rad, Hercules, CA, USA).

### Library Preparation and scRNAseq

Single-cell cDNA library preparation and sequencing were conducted at the Genomics Resource Center core facility, Institute for Genome Sciences, University of Maryland School of Medicine (Baltimore, MD). cDNA libraries were prepared using Chromium Next GEM Single Cell 3′ GEM, Library & Gel Bead Kit v3.1 (10×Genomics, Pleasanton, CA, USA) on the 10×Genomics Chromium Controller according to the manufacturer's manual for a target of 4000 cells per sample. After barcoded cDNAs were obtained, the libraries were sequenced with the Illumina NovaSeq6000 platform using a high-output flow cell to obtain 100-bp paired-end reads at a depth of 25 000 total reads per cell for each respective library.

### Alignment

The raw single-cell sequencing data were processed using the 10×Genomics Cell Ranger v6.0.2 software (10×Genomics) (14). The reads were aligned to the GRCz11/danRer11 (Ensembl release 106; GCA_000002035.4) zebrafish genome. Gene annotation was filtered using the “mkgtf” command to include only protein-coding genes (*− attribute = gene_biotype:protein_coding*). The standard protocol of the “count” function was used with default parameters for preliminary analyses, including quality control for reads, genome alignment using Spliced Transcripts Alignment to a Reference, barcode recovery, and quantification of gene expression to generate a gene-by-cell expression count matrix.

### Initial Data Processing

The following data processing and visualizations were performed using R v4.1.1 and v4.3.2 (15). Seurat v4 R package was utilized for downstream analyses (16). Cells with less than 500 and more than 50 000 Unique Molecular Identifiers, fewer than 500 detected genes per cell, and more than 10% mitochondrial genes per cell were excluded, as they generally represent poor-quality cells or empty droplets or doublets. This quality control resulted in 2194 cells and 25 432 genes for WT and 4794 cells and 25 432 gene for the *gnrh3*^−/−^ sample. The filtered single-cell data sets from different genotypes were normalized using default settings of the “NormalizedData” function. Highly variable genes were then identified by “FindVariableFeatures” to select the top 2000 variable genes in each sample. The data sets from WT and *gnrh3*^−/−^ were integrated using canonical correlation analysis to suppress variance between the libraries, and the integrated data was utilized for further analyses.

### Cell Type Identification

The top 2000 variable features were used as input for principal component analysis using the “RunPCA” function. Identification of cell types was performed using the top 20 principal components and a resolution of 0.4 using the Louvain algorithm with “FindNeighbors” and “FindClusters” functions. In order to identify genes enriched in individual cell types, a Wilcoxon rank-sum test was applied for each cluster against the remaining clusters using the “FindAllMarkers” function with the following parameters: only.pos = TRUE, min.pct = 0.25, logfc.threshold = 0.25. The cell type identity was assigned using manual annotation using known pituitary marker genes (12, 17, 18). The Zebrafish Information Network (https://zfin.org) was referenced for nomenclature for the zebrafish gene names. If the appropriate gene names were not available in the Zebrafish Information Network database, we used the Ensembl stable gene IDs from GRCz11 (Ensembl release 106) zebrafish genome assemble database. We projected the integrated data into low-dimensional uniform manifold space on the top 20 principal components and visualized each cell type. The similarity of the same cell types between the WT and *gnrh3*^−/−^ samples in the integrated data was evaluated using the calculation of Pearson correlation coefficient using average expressions of genes in each cell type.

### Differential Gene Expression and Pathway Enrichment Analysis

Identified differentially expressed genes (DEGs) were identified for a WT cell type against the same cell type in *gnrh3*^−/−^ by applying the Model-based Analysis of Single-cell Transcriptomics test (19) in LH and FSH gonadotropes through the “FindMarkers” function, and false discovery rate-adjusted *P*-values and fold-change values were acquired for all detected genes. All TF gene information in zebrafish was obtained from a TF database: AnimalTFDB (20). Gene set enrichment analysis was performed to estimate the enriched pathways in each cluster using iDEA R package (21) with default workflow and parameters. The Gene Ontology (22-25), MSigDB (24, 25), KEGG (26), and Reactome (27) databases were used for referencing pathway definitions.

### Ablation of Gnrh3 Neurons

Gnrh3 + cell-specific ablation was performed by a nitroreductase-mediated method on 5-month-old WT and *Tg(gnrh3:Gal4ff; UAS:nfsb-mCherry)* females, by exposure to metronidazole (Mtz) in water, following a previously described protocol (11). Briefly, 3 experimental groups of females were prepared: (1) Transgenic (Tg)-Mtz: *Tg(gnrh3:Gal4ff; UAS:nfsb-mCherry)* fish with 2 mM Mtz in vehicle (0.2% dimethyl sulfoxide in system water); (2) Tg-control (Cont): *Tg(gnrh3:Gal4ff; UAS:nfsb-mCherry)* fish with vehicle; and (3) WT-Mtz: WT siblings (no transgene expression) with 2 mM Mtz in vehicle. To obtain females with the same reproductive stage start point, the evening before the beginning of the treatment, females were set for spawning with WT males according to routine protocol for zebrafish spawning. Females that spawned were selected for the experiment that began the day of the spawning. The treatments were performed during the 10 hours night phase and replenished daily. On day 14 of the treatment, pituitaries were dissected and fixed in 4% paraformaldehyde overnight at 4 °C.

### Whole-mount In Situ Hybridization

*lhb* and *fshb* expressions in the pituitary were detected by whole mount in situ hybridization chain reaction. Probes and fluorescently conjugated amplifiers were purchased from Molecular Instruments (Pasadena, CA, USA). The manufacturer designed 2 40 bp/gene *lhb* and *fshb* probes based on published cDNA sequences, GenBank accession AY714132.1 and AY714131.1, respectively. *lhb* and *fshb* probes were designed to be detected with amplifiers conjugated to 488 and 546 nm fluorophores, respectively. The fixed intact pituitaries were washed with PBS, treated with 1 µg/mL proteinase K (Invitrogen), and post-fixed with 4% paraformaldehyde for 30 minutes followed by prehybridization at 37 °C for 2 hours in hybridization buffer. After probes were added to a final concentration of 2 pmol, the tissues were hybridized at 37 °C overnight. The signals were obtained using the hybridization chain reaction whole-mount kit (Molecular Instruments Inc.), following the manufacturer's protocol. After mounting, entire pituitaries were analyzed with a Leica SP8 confocal microscope (Leica Microsystems, Wetzlar, Germany) set to capture 45 × 2 µm z-stacks. A maximum intensity projection was then generated, and all *lhb^+^* and *fshb^+^* cells were manually counted using ImageJ software. To ensure accuracy, only *lhb^+^* and *fshb^+^* cells that corresponded exactly to those identified by Hoechst 33342 staining were included in the count (28).

## Results

### Lack of Gnrh3 Retains Cell Type Distribution in the Pituitary

**Figure 1.**
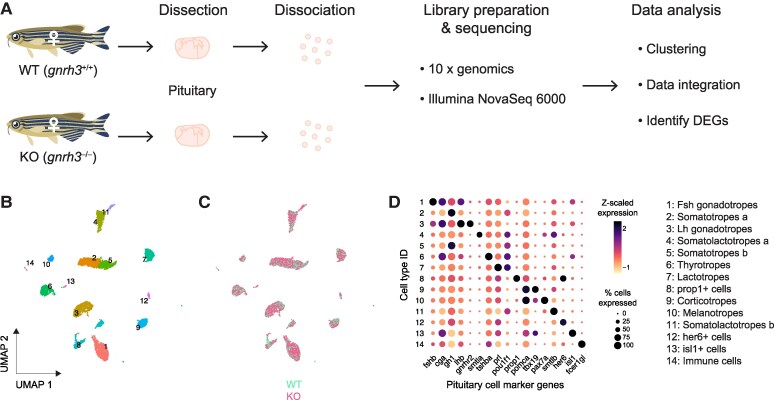
Identification of major pituitary cell types in wild-type and *gnrh3*^−/−^ sexually mature female zebrafish pituitary. (A) Schematic presentation of single-cell RNA sequencing workflow. (B) UMAP representation of molecularly defined distinct cell types in the pituitaries. (C) UMAP representation of pituitary cell types by genotype. (D) Dot plot classifying pituitary cell types by known marker gene expression. The color and size of the dots represent the z-scaled average gene expression level and the proportion of cells expressing the gene, respectively. Abbreviation: UMAP, uniform manifold.

To characterize the transcriptomic profiles of the sexually mature female pituitary and determine cell type heterogeneity between WT and *gnrh3*^−/−^, we performed scRNAseq (Fig. 1A). A total of 120 652 910 and 121 906 443 raw reads were obtained from which 95.3% in the WT sample and 97.1% in the *gnrh3*^−/−^ sample were mapped to the reference genome, respectively. The total number of 2194 cells in the WT sample and 4794 cells in the *gnrh3*^−/−^ sample passed quality control criteria, and 25 432 genes were detected in total.

We integrated the sequencing data from the WT and *gnrh3*^−/−^ samples and performed clustering, which identified 14 cell types (Fig. 1B and 1C). In reference to the known pituitary cell marker genes (12, 18), 10 known hormone-secreting endocrine cell types were classified as lactotropes (*prl*+), somatolactotropes (*smtla* + and *smtlb*+), somatotropes (*gh*+), thyrotropes (*tshb* + and *cga*+), FSH gonadotropes (*fshb* + and *cga*+), LH gonadotropes (*lhb* + and *cga*), melanotropes (*pomca*+), and corticotropes (*pomca*+). Two potential progenitor clusters, *isl1* + cells (*isl1* + and *cga*+) and *prop1* + cells (*prop1* + and *her6*+), as well as endothelial cells (*plvapa* + and *her6*+) and potential immune cells (*fcer1gl*+) were identified [Fig. 1D; Supplementary Table S1 (29)]. Interestingly, LH and FSH gonadotropes also expressed lower levels of the other gonadotropin beta subunit (Fig 1D and Table 1), and LH gonadotropes also contained minute levels of hormone genes like *pomca*, *prl*, and *gh1* (Fig 1D). The identified classical cell types were aligned with those previously reported for the adult male zebrafish pituitary (12).

**Table 1.** Single-cell RNA sequencing *lhb* and *fshb* transcript levels in LH and FSH gonadotropes

Differential gene expression of *lhb* and *fshb* in LH and FSH populations		
Gene	FSH gonadotropes	LH gonadotropes
*fshβ*	197.41588	5.04349
*lhβ*	53.49077	691.83146

Percent of LH and FSH cells expressing each *lhb* and *fshb*		
Gene	% Cells expressing each gene	ID
*lhβ*	80.87805	FSH gonadotropes
*fshβ*	97.85366	FSH gonadotropes
*lhβ*	100	LH gonadotropes
*fshβ*	52.33533	LH gonadotropes

### Gene Expression Profile in Zebrafish Gonadotropes

#### Receptors

**Figure 2.**
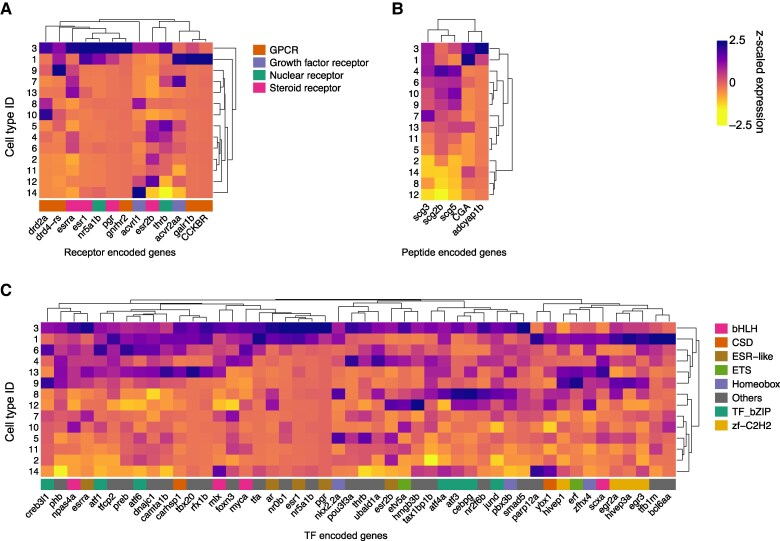
Molecular factors potentially regulating LH and FSH secretion in zebrafish females. Heatmaps of z-scaled expression centered at 0 for individual comparisons between cell types for genes encoding (A) receptors, (B) peptides, and (C) transcription factors.

In order to identify specific regulators of gonadotropins, the present study focused on highly enriched genes encoding receptors in LH and FSH gonadotropes, compared to all other cell types (Fig. 2A). Ten receptor encoding genes were highly expressed in LH gonadotropes: dopamine receptor D2a (*drd2a*), dopamine receptor D4 related sequence (*drd4-rs*), estrogen-related receptor alpha (*esrra*), estrogen receptor 1 (*esr1*), nuclear receptor subfamily 5 group A 1b (*nr5a1b,* also known as *sf-1*), progesterone receptor (*pgr*), gonadotropin-releasing hormone receptor 2 (*gnrhr2*), activin A receptor like type 1 (*acvrl1*), and thyroid hormone receptor beta (*thrb*). Estrogen receptor type 2b (*esr2b*) has also been detected in LH gonadotropes, but its expression level was lower than the top 10 receptors. Six genes were significantly enriched in FSH gonadotropes: *drd-4*, *esr1*, steroidogenic factor 1 (*nr5a1b*), activin A receptor type 2Aa (*acvr2aa*), galanin receptor 1b (*galr1b*), and predicted cholecystokinin receptor like (*Cckrb*, *ENSDARG00000076824*). Among the 4 paralogous genes encoding GnRH receptors in zebrafish (*gnrhr1*, *gnrhr2*, *gnrhr3*, and *gnrhr4*) (30), only *gnrhr2* was identified in our pituitary dataset, and it was uniquely expressed in LH gonadotropes.

#### Endocrine regulators

In an effort to detect potential autocrine or paracrine regulators for LH or FSH gonadotropes, we examined the expression profile of peptide encoding genes in these cell types (Fig. 2B). Remarkably, both *lhb* and *fshb* were expressed in both gonadotrope types. Fifty-two percent of LH gonadotropes coexpressed *fshb* with a ratio of 1/138 *fshb/lhb*, and 81% of FSH gonadotropes expressed *lhb*, albeit 3.7 times lower than that of *fshb* (Table 1). Furthermore, predicted glycoprotein hormones alpha chain-like (*CGA*, ENSDARG00000103345) and adenylate cyclase activating polypeptide 1b (*adcyap1b*), secretogranin III (*scg3*), and secretogranin V (*scg5*) were enriched in both LH and FSH gonadotropes.

#### Transcription factors

In our quest to reveal potential regulatory pathways within LH and FSH gonadotropes, we profiled the gene expression of TFs in those cell types (Fig. 2C). A set of 48 TFs demonstrated significant enrichment in both LH and FSH gonadotropes, including members from subclasses such as 3 basic helix-loop-helix (bHLH), cold shock protein, estrogen receptor (ESR)-like, ETS, homeobox, zebrafish bZIP, zebrafish C2H2, and others. Half of the identified enriched TFs were common to both LH and FSH gonadotropes, while 10% were unique to LH and 40% exclusive to FSH gonadotropes.

### Identification of Functional Differences Between the Gonadotropes of WT and Gnrh3^−/−^

**Figure 3.**
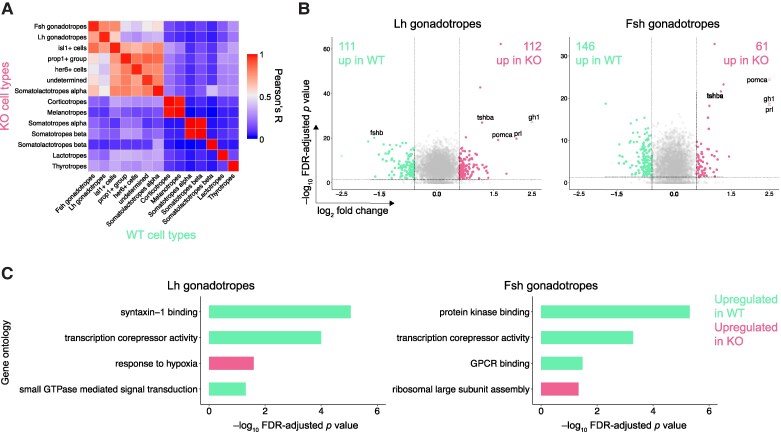
Major differences between WT and *gnrh3^−/−^* pituitaries. (A) Heatmap illustrating Pearson's correlations in gene expression between the WT and *gnrh3^−/−^* cell types. (B) Volcano plots representing differentially expressed genes, DEGs, defined by *P* < .05 and fold change > 1.5 in the *gnrh3^−/−^* LH gonadotropes (left) and FSH gonadotropes (right) vs those of the WT pituitary sample. (C) Bar plots representing significant enrichment of DEGs in specific Gene Ontology categories. ***P < .001. Abbreviations: DEG, identified differentially expressed gene; WT, wild-type.

To identify a possible disparate modulation of gonadotropes in the absence of Gnrh3, we compared the gene expression between WT and *gnrh3*^−/−^ gonadotropes and DEGs that exhibited a ≥ 1.5-fold change and passed a false discovery rate-corrected *P*-value < .05 [Supplementary Table S2 (31)]. An average of the gene expression levels within individual clusters undertaken through Pearson correlation coefficient analysis showed similar patterns of gene expression within identical cell types between WT and *gnrh3*^−/−^, featuring Pearson's R values between 0.96 and 0.99 (Fig. 3A).

We next focused on DEGs within LH and FSH gonadotropes. This revealed 111 and 112 DEGs being upregulated in LH gonadotropes, alongside 146 and 61 DEGs being upregulated in FSH gonadotropes in WT and *gnrh3*^−/−^, respectively. Notably, 9 DEGs were found to be upregulated in both *gnrh3*^−/−^ LH and FSH gonadotropes, encompassing genes that encoded pituitary hormones like *gh1*, *prl*, *pomca*, and *tshba*. One hundred and 101 DEGs are uniquely upregulated in WT and *gnrh3*^−/−^ LH gonadotropes, respectively, but not in FSH gonadotropes (Fig. 3B and 3C). Interestingly, *fshb* displayed significantly elevated expression in WT LH gonadotropes compared to its expression in *gnrh3*^−/−^.

To elucidate the molecular and cellular pathway shifts in *gnrh3*^−/−^, we deployed gene set enrichment analysis for Gene Ontology pathways (Biological Process and Molecular Function), based on summary statistics derived from the DEG identification. In LH gonadotropes, syntaxin−1 binding, transcription corepressor activity, and small GTPase-mediated signal transduction-related pathways were upregulated in WT, while the genes relevant to a response to the hypoxia pathway were more enriched in *gnrh3*^−/−^. In the case of FSH gonadotropes, protein kinase binding, transcription corepressor activity, and GPCR binding pathways were upregulated in WT, whereas the ribosomal large subunit assembly was primarily upregulated in *gnrh3*^−/−^ (Fig. 3D).

### Effect of Gnrh3 Neurons Chemogenetic Ablation on Pituitary Fshb and lhb Positive Cell Types

**Figure 4.**
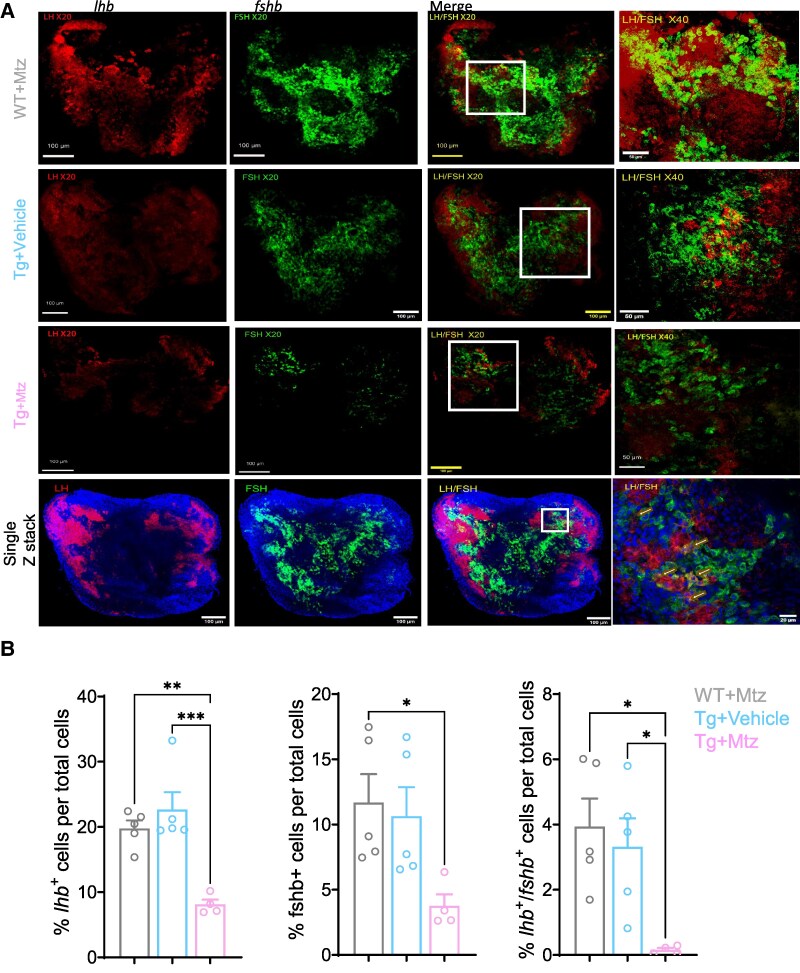
Depletion of GnRH3 neurons leads to reduced gonadotropes number in mature female pituitaries. WT females and *Tg(gnrh3:Gal4ff; UAS:nfsb-mCherry)* treated with either vehicle or Mtz for 14 days. Pituitaries were subjected to double labeling in situ hybridization followed by cell counting. (A) Pituitary of representative WT with Mtz (upper panel), Tg with vehicle (second panel from top), and Tg with Mtz (third panel from top) treated females. Fourth panel depicts a single 20 µm Z-stack image of WT pituitary stained with Hoechst 3342. From left to right: *lhb* mRNA (red), *fshb* mRNA (green), merged image to analyze colocalization of *lhb* and *fshb*, and a magnification of the rectangular area depicting region. (B) Percentage of *lhb*^+^, *fshb*^+^, and *lhb*^+^/*fshb*^+^ colocalized cells in the proximal pars distalis (PPD) counted in the photographed pituitaries (WT-Mtz and Tg-Cont, n = 5, Tg-Mtz, n = 4). Results are presented as mean ± SEM. Statistical significances were tested by one-way ANOVA followed by Tukey multiple comparison test. *P < .05, **P < .01, ***P < .001, ****P < .0001. Abbreviations: Mtz, metronidazole; Cont, control; WT, wild-type; Tg, transgenic.

In order to examine whether the absence of Gnrh3 leads to a decrease in *fshb* expression observed in the scRNA-seq, we implemented a conditional ablation of Gnrh3 neurons followed by in situ hybridization to quantify the number of *lhb +* and *fshb +* cells. Depletion of Gnrh3 neurons using exposure to Mtz for 14 nights resulted in a significant decrease of *fshb* + cells from 12% to 4% of the total cell number in the pituitary (*P* ≤ .05). Similarly, *lhb* positive cells showed a decreased prevalence from about 19% to 8% (*P* ≤ .0001) and *lhb+;fshb +* from about 4% to nearly 0% (*P* ≤ .05) (Fig. 4A). These images also demonstrated that the majority of the *lhb+;fshb +* are found in, but not limited to, the borderline between *lhb + and fshb +* gonadotropes (Fig. 4B).

## Discussion

scRNAseq and single nucleus RNAseq techniques were employed to transcriptionally define cell types and gene transcripts enriched in specific populations in pituitary cells from teleost to mammals (12, 13, 18, 30, 32-34). To reveal the roles of Gnrh3 in the pituitary of female zebrafish, we performed scRNAseq on pituitaries of sexually mature WT and *gnrh3^−/−^* females to identify cell-specific transcriptional changes and deduce potential signaling pathways involved in LH and FSH synthesis/secretion. We identified 14 cell types in the pituitaries of both lines, of which 10 cell types represented the 6 classical hormone-producing endocrine cell populations and 2 potential progenitor cells; the rest were endothelial and immune cells (Fig. 1C). The presence of all tetrapod hormone-releasing endocrine cell types (12, 13, 18, 30, 32-34) was confirmed in female zebrafish pituitary and was identical to that described for male zebrafish and medaka pituitary cell populations (12, 13). Our data corroborate the notion of teleost separate FSH and LH gonadotropes; however, unlike the distinct LH and FSH gonadotropes in teleosts, both gonadotrope types expressed a reduced level of the counterpart gonadotropin. Overall, our transcriptome analysis suggests that the loss of function of the *Gnrh3* gene does not affect the gross pituitary endocrine cell type composition in zebrafish females, although subtle changes in *fshb* expression were noted.

Previous studies have described the presence of cells expressing multiple endocrine hormones (termed “multihormonal cells’) in teleosts and mammals (35-40). In medaka pituitary, cells that coexpress *lhb* and *fshb*, *fshb* and *tshb*, and *sl* and *lhb* were identified (39, 40). In mammals, such multihormonal cells are typically associated with pituitary plasticity, particularly with a transitional state (35) and typically express PROP paired-like homeobox 1 (*PROP1*), SRY-related HMG box transcription factors, SOX2 and SOX9 (41-43). *Sox2* is recognized as a marker gene for pituitary progenitor cells in mice, humans (44), and medaka (45). A previous study in zebrafish has identified *sox2 +* cells in the anterior pituitary at 42 to 72 hours postfertilization (46), indicating a temporal expression pattern during development. However, it remains unclear whether *sox2* serves as a progenitor marker in the adult zebrafish pituitary. A prior single-cell RNA sequencing study of male zebrafish pituitary did not recognize *sox2* as a progenitor marker; instead, it focused on *her6* and *isl1* (12), and thus we utilized these genes as progenitor markers in our study. Nevertheless, our data reveal that *sox2* expression is enriched in the *prop1+*, corticotropes, melanocorticotropes, and *her6 +* cells, suggesting that potential progenitor cell types were not misclassified [Supplementary Fig. S1 (47)]. The cells expressing *sox2* in the pituitary of medaka, which were found in the border of the neurohypophysis and adenohypophysis with a few cells distributed in the adenohypophysis (45), may be corticotropes and melanocorticotropes, like in zebrafish.

The scRNAseq data reveal that all endocrine cells express more than 1 gene encoding pituitary hormones, indicating that the phenomenon of multihormonal expression is conserved across species. The multihormone cells in the pituitary may originate from immature progenitor cells with multipotent properties. These cells could enter a transitional state where they simultaneously produce more than 1 hormone-encoding mRNA (43, 48), or they may be in a stage of transdifferentiation, transitioning from 1 specific hormone-producing cell type to another (49). Another possibility is that certain cells possess a dormant capacity to express multiple hormones, which can be activated by neuroendocrine or endocrine signals (40). The latter scenario better explains the disappearance of *fsh+/lh +* cells in the pituitary of Gnrh3 neuron-ablated females (Fig. 4) and the upregulation of genes expressing other hormones in the *gnrh3^−/−^* female pituitaries (Fig. 3A). Still, however, the function of the *fsh^+^/lh^+^* cells in the pituitary remains elusive due to the lack of *cga* and receptor genes or any other known cell marker gene transcripts. Whether the genes encoding nonprimary hormone in the endocrine cells contribute to their functional roles and when those systems are active needs to be studied.

Although the hypothalamic Gnrh3 and its neurons are dispensable for regulating LH secretion from the pituitary (10, 11), we found that out of the 4 GnRH receptor type genes (*gnrhr1-4*) detected by PCR in the pituitary of zebrafish (50), only *gnrhr2* transcript was detected in the pituitary of mature female and was specifically expressed in LH gonadotropes (Fig. 2A). This is either unique to mature female pituitary, or the transcript levels of the other 3 is below the detection limit of this procedure. Gnrhr2 belongs to the nonmammalian type III GnRH receptor and exhibits equal potency with both native ligands, Gnrh2 and Gnrh3 (30, 51). Nevertheless, the high transcript level of Gnrhr2 in LH gonadotropes combined with the massive projections of Gnrh3 neurons into the proximal pars distalis (PPD) suggests a functional role for Gnrh3 in these cells. In fact, a recent study has shown that LH gonadotropes exhibit a strong and slow calcium rise in response to Gnrh3 analog using brain-pituitary ex vivo preparation in zebrafish (52), clearly demonstrating that Gnrh3 activates LH gonadotropes. Yet, the effect of Gnrh3 on LH secretion in zebrafish is still controversial due to negligible changes in LH levels in the in vitro setting, intraperitoneal injection, and intracerebroventricular injection of Gnrh3 solely or with a dopamine antagonist (53, 54). Notably, when tested in vitro, Gnrh3 stimulation of zebrafish Gnrhr2 did not induce cAMP signaling (50). Since cAMP activation is required for LH secretion and the preovulatory LH surge in rodents (53), it may well also be the case in fish, thus supporting the idea that Gnrh3 is not a main inducer of LH synthesis and secretion in zebrafish (11). We have, however, observed that adenylate cyclase-activating polypeptide 1b (*adcyap1b*) expression is significantly higher in WT LH gonadotropes compared to *gnrh3*^−/−^, indicating the higher levels of cAMP in LH gonadotropes of WT pituitary are somehow connected to Gnrh3 or to other hormones or growth factors that are expressed on LH cells.

As mentioned earlier, our scRNAseq did not detect *gnrhr* transcript in FSH gonadotropes (Fig. 2A). This finding is similar to what is found in the medaka (13) but different from the case of tilapia, where different GnRH receptor isoforms were found in LH and FSH gonadotropes (55). Instead, the transcripts of 2 other neuropeptide receptors were enriched in FSH gonadotropes: Cckrb and Gal1rb (Fig. 2A). Cck has recently been shown to activate FSH gonadotropes and induce FSH secretion in zebrafish and medaka (52, 56). Gal1rb is predicted to be activated by galanin (57), unlike Gal2r, which is activated by Spexin (58).

If indeed both LH and FSH gonadotropes express low levels of the counterpart gonadotropin, it could lead to a scenario where the activation of FSH gonadotropes results in the release of both LH and FSH. Similarly, activating LH gonadotropes could stimulate FSH release. Although this has yet to be proven, the proposed stimulatory effect of Gnrh3 on *fshb* in LH gonadotropes, combined with the larger number of LH gonadotropes compared to FSH gonadotropes, suggests that sufficient levels of FSH may be released to promote ovarian development in female zebrafish. Given that previous studies have shown that LH and FSH gonadotropes exclusively express either *lhb* or *fshb*, respectively (59, 60), these findings should be interpreted with caution. There remains the possibility that the dispersion process induced transcriptional changes that do not occur when the cells are attached (36, 59).

It is well known that in mammals gonadotropin secretion, as well as its transcription, are dependent on GnRH pulse frequency (61). Similar to mammals, the expression levels of *lhb* in the pituitary of intact and ovariectomized *gnrh1*^−/−^ female medaka were significantly lower than that of WT (6), implicating the importance of Gnrh1 in the transcriptional regulation of gonadotropin subunits. In contrast, our previous work has shown that the expression level of *lhb* or *fshb* is unchanged in the absence of Gnrh3 in LH or FSH gonadotropes of zebrafish (9, 10). Together, the role of the hypophysiotropic Gnrh3 in zebrafish is likely different from other species in the context of regulation of gonadotropin synthesis and secretion. Nevertheless, since Gnrh3 is secreted in a pulsatile manner (62), our RNA-seq data representing a single timepoint cannot completely rule out the possibility of Gnrh3 playing a role in the regulation of the gonadotropins.

Other than the effect of Gnrh3, other factors notably affect LH and FSH gonadotropes, some specific and some common to both cell type in the mature female pituitary. Gonadal steroids act on both gonadotropes thorough the receptors *esr1* and *pgr* and the transcription factor nra1b/sf-1. Dopamine is a prominent regulator of reproduction in cyprinids including carp, goldfish, and zebrafish (54, 63, 64) and likely acts on the gonadotropes through 2 isoforms of dopamine receptors: drd2 in LH gonadotropes and drd4 in FSH gonadotropes. Both drd2 and drd4 belong to the family of inhibitory D2-like dopamine receptors, which are involved in reducing cyclic AMP levels and modulating neuronal activity. It was shown that LH gonadotropes in zebrafish express 3 types of drd2 receptors (a-c), which may all be involved in the dopaminergic inhibitory action on LH secretion (54). The *drd4* gene is recognized for its link to behavioral traits in various animal taxa, including fish (65). Its presence on FSH cells suggests that the dopaminergic effect on FSH, unlike LH, is likely mediated through *drd4*. The gonad-derived peptide activin seems to act on both gonadotropes via 2 different receptors, *acvrl1* and *acvrl2aa*, reflecting its stimulatory and inhibitory effect on LH and FSH gonadotropes, respectively, as reported for goldfish and zebrafish (66).

The DEGs encoding for TFs were investigated in LH gonadotropes of sexually mature female zebrafish. Thirty-seven significantly highly expressed genes encoding for TFs in LH gonadotropes were identified. Among those, 7 genes, including *atf3*, *creb3l1*, and *jun*, belong to the bZIP family. bZIP and bHLH are known to be key regulators of *nr5a1*/*sf-1* gene expression in the pituitary gonadotropes (67). Nr5a1 regulates steroid metabolic enzymes and expression of the genes involved in various cellular processes (68). Pituitary-specific knockout of Nr5a1 in mice resulted in depleted *lhb* and *fshb* levels and infertility (69). We have observed that the *nr5a1b* expression is unchanged between WT and *gnrh3*^−/−^ in LH gonadotropes. This supports the theory that Nr5a1b transcriptional regulation plays a key role in controlling *lhb* and *fshb* expression in zebrafish. Together with the known ESR-like family members found in LH gonadotropes, these results suggest that estrogen is a potential major regulator of LH gonadotropes.

Significantly high expression of ESR genes (*esr1*, *esrra,* and *esr2b*) in LH gonadotropes of female zebrafish support the notion that estrogen directly regulates LH secretion. It has been previously shown that E_2_ directly acts on the pituitary and induces Lhb protein synthesis in pubertal female zebrafish (70). Congruently, whole pituitary incubation with E_2_ in vitro illustrates that E_2_ is a potent inducer of Lh secretion from mature female pituitary, which exhibits a much stronger elicitation than Gnrh3 (71). These findings disclose the presence of a direct pituitary-gonadal estrogen positive feedback mechanism in female zebrafish. Indeed, it has been demonstrated that estrogen receptors bind estrogen-responsive element (ERE) regions on the *lhb* promoter (70). Direct effects of estrogen on LH synthesis and secretion have also been reported in mammals (72-74).

The inherited lack of hypophysiotropic GnRh in zebrafish upregulated *gh1*, *tshb*, *pomca*, and *prl* and downregulated *fshb* compared to the WT. Gnrh1 induces changes in the chromatin at the promoter of the *Cga*, *Lhb*, and *Fshb* genes in mice through histone phosphorylation, acetylation, and citrullination that possibly facilitate nucleosome eviction and gene transcription (75-78). However, *cga* and *lhb* expression levels do not change in zebrafish LH gonadotropes of *gnrh3^−/−^*, suggesting that, unlike in other vertebrates, zebrafish Gnrh3 modulates *fshb* expression but not *cga* and *lhb* in Lh gonadotropes. The results also suggest that the promoter of the *gh1*, *tshb*, *pomca*, and *prl* genes are amenable to respond to Gnrh3 signaling. A series of other changes were observed in *gnrh3*^−/−^ LH gonadotropes, all indicating a role for Gnrh3 in suppressing a set of genes, thus contributing to the specialization of LH gonadotropes. On the other hand, except for the protein secretion-related pathway, potential pathways involved in LH secretion, including *esrra* and *esr1* mediated signaling, are unaffected. This suggests that the lack of Gnrh3 may increase the responsiveness of LH gonadotropes to estrogen at the nuclei level.

In order to establish whether Gnrh3 and/or its neurons play a role in the organization of the pituitary, a chemogenetic conditional depletion of Gnrh3 neurons was performed followed by double-labeled in situ hybridization for *lhb* and *fshb* in the pituitaries (Fig. 4). Gnrh3 neuronal depletion resulted in reduced numbers of LH and FSH gonadotropes, as well as of *fshb*+*/lhb* + cells. These results are consistent with the downregulation of *fshb* seen in LH gonadotropes in *gnrh3*^−/−^ pituitaries; still, they do not explain the reduction in the number of both gonadotropes and *fshb*+*/lhb* + cells. The mechanism by which the pituitary is affected by Gnrh3 neurons is unknown and may lie in the fact that the entire neurons are removed by the ablation. The lack of changes observed in *gnrh3*^−/−^ pituitaries with the lack of Gnrh receptor in FSH gonadotropes supports the notion that the downregulation of *lhb* + and *fshb* + is caused by the absence of another factor in Gnrh3 neurons.

**Figure 5.**
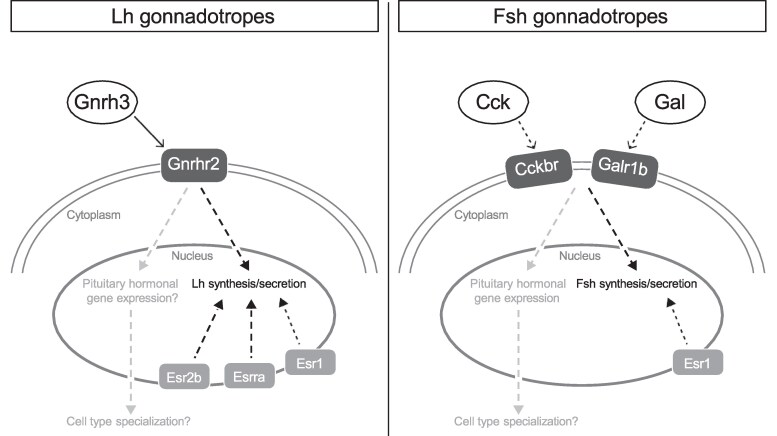
Proposed functional regulatory pathways of LH and FSH gonadotropes in adult female zebrafish. Gnrh3 directly acts on LH gonadotropes through Gnrhr2 to regulate gene expression of various non-*Lhb* hormone encoding genes, including *fshb*, by that contributing to LH-gonadotrope specialization. Alternatively, the upregulation of the nonprimary hormones in LH gonadotropes occurs during the preparation of the cells for single-cell RNA sequencing and is denoted by the question mark next to gene expression. The estrogenic pathway seems prominent in LH gonadotrope and manifested via 3 different estrogen nuclear receptors that facilitate LH synthesis and secretion. The expression of the receptors *galr1b* and *cckrb*, but not *Gnrhr*, in FSH gonadotropes infers a non-GnRH regulation of FSH synthesis and secretion. The estrogenic pathway is less prominent in FSH gonadotropes and is executed only via 1 nuclear receptor, Esr1.

Altogether, our results demonstrate that hypophysiotropic Gnrh3 acts on LH gonadotropes via the GnRH receptor to regulate the expression of genes encoding pituitary hormones. Gnrh3 may therefore play a role in the specialization of endocrine cell types in the pituitary. However, the upregulation of other hormone genes in LH gonadotropes of *gnrh3^−/−^* fish should be interpreted with caution (Fig. 5). Collective data from our studies, alongside the fertility phenotype of *gnrh3/gnrh2^−/−^* zebrafish (9, 10), fertility outcomes following Gnrh3 neuron ablation (11), and the negligible changes in LH levels in various experimental settings, including in vitro studies, intraperitoneal injection, and in vivo intracerebroventricular injection of Gnrh3 alone or with a dopamine antagonist (53, 54), suggest that Gnrh3 does not significantly affect LH levels. Alternatively, LH secretion and synthesis are likely regulated by multiple factors, with E_2_ being an established regulator (70), in female zebrafish.

## Data Availability

The scRNAseq data generated in this study have been deposited in the Gene Expression Omnibus under accession number GSE272806.
