# Correction to: “GnRH—Gonadotropes Interactions Revealed by Pituitary Single-cell Transcriptomics in Zebrafish”

**DOI:** 10.1210/endocr/bqaf098

**Published:** 2025-06-03

In the above-named article by Tanaka S, Yu Y, Levavi-Sivan B, Zmora N, and Zohar Y (*Endocrinology*. 2024; 165(12); doi: 10.1210/endocr/bqae151), there was an error in the Correspondence section.

In the published article, in the Correspondence section, author Yonathan Zohar’s correspondent address was incorrectly published as, “Department of Animal Sciences, The Robert H. Smith Faculty of Agriculture, Food, and Environment, The Hebrew University of Jerusalem, Rehovot 76100, Israel.” In the corrected article, Yonathan Zohar’s correspondent address has been updated to read, “Institute of Marine & Environmental Technology, Department of Marine Biotechnology, University of Maryland Baltimore County, Baltimore, MD 21202, USA.”

The article has been corrected online.

doi: 10.1210/endocr/bqae151

